# Posterior Dislocation of the Hinge-Post Extension in a Rotating Hinge Total Knee Prosthesis

**DOI:** 10.1155/2013/756538

**Published:** 2013-09-23

**Authors:** Givenchy Manzano, Ran Schwarzkopf

**Affiliations:** Department of Orthopaedic Surgery, Joint Replacement Service, University of California Irvine Medical Center, 101 The City Drive South, Pavilion III, Building 29, Orange, CA 92868, USA

## Abstract

The rotating hinge knee prosthesis is a popular intervention in patients lacking stability with highly constrained total knee arthroplasty. Despite improvements in design, nonmechanical and mechanical complications continue to be a problem. Dislocation of the hinge has been widely described, mainly due to the component fracture. Few reports describe isolated dislocation of the rotating stem. We report a case of isolated disengagement of the rotating hinge mechanism, due to severe flexion gap imbalance, leading to subsequent posterior dislocation of the hinge and anterior knee dislocation, in a patient with a history of multiple total knee arthroplasty revisions. This case suggests the importance of the soft tissue balancing, the adequate patellar tracking, and use of a long cylindrical, minimally tapered rotating stem in hinge arthroplasty to minimize hinge dislocation.

## 1. Introduction

The rate of revision total knee arthroplasty in the United States has been steadily increasing over the years [1]. Rotating hinge knee prostheses are used when less constrained knee implants fail to provide stability. Initially designed as an evolution of the fixed hinge knee implant to reduce the risk of aseptic loosening, its use has been extended for use in patients after radical tumor resection about the knee as well as in complicated primary, revision, and salvage knee reconstruction [2]. Modern rotating hinge implants evolved in an attempt to avoid aseptic loosening and component fractures seen in the first-generation fixed hinge knee prostheses by allowing motion in more than one plane to decrease the high stresses on the articulation and at the bone cement interface [2]. The effects of these improvements are reflected in reports of rotating hinge arthroplasty survival rates of 89.2%–96.1% after 6 to 20 years [3, 4]. Despite their evolution in design, infection, loosening, patellar instability, component fracture, and implant dislocation continue to be a common problem [5–8]. 

Failure of rotating hinge arthroplasty due to hinge dislocation has been widely published in the literature [7–12]. Most cases reported describe failure due to component fracture with an incidence of 2% to 10% [5, 7, 8, 13, 14]. C. J. Wang and H. E. Wang reported breakage of the polyethylene bearing bush of the femoral channel component on the metallic tibial stud within 5 months from implantation of an Endo-Model arthroplasty (Waldemar Link GmbH & Co., Hamburg, Germany) [9]. In 2008, Pacha-Vicente et al. described breakage of the antidislocation component in an Endo-Model implant, causing hinge dislocation in 2 patients [12]. In 2011, Schwarzkopf et al. reported 2 cases of fracture of the tibial post in a DePuy S-ROM prosthesis (DePuy, Warsaw, IN USA). Subsequently, Friesenbichler et al. reported fracture of the tibial metal yolk in patients with an LPS knee system (DePuy, Warsaw, IN USA) [7]. Recently, Chuang and colleagues reported failure of a rotating hinge megaprosthesis due to breakage of the tibial polyethylene stopper [14]. Despite these reports of hinge failure, only 2 reports of atraumatic disengagement of the rotating hinge stem have been described in the literature [10, 11]. Here, we report a case of atraumatic disengagement of the rotating stem due to opening of the flexion gap, leading to subsequent posterior dislocation of the hinge and anterior knee dislocation in a patient with a history of multiple total knee arthroplasty revisions.

## 2. Case Study

**Figure 1:**
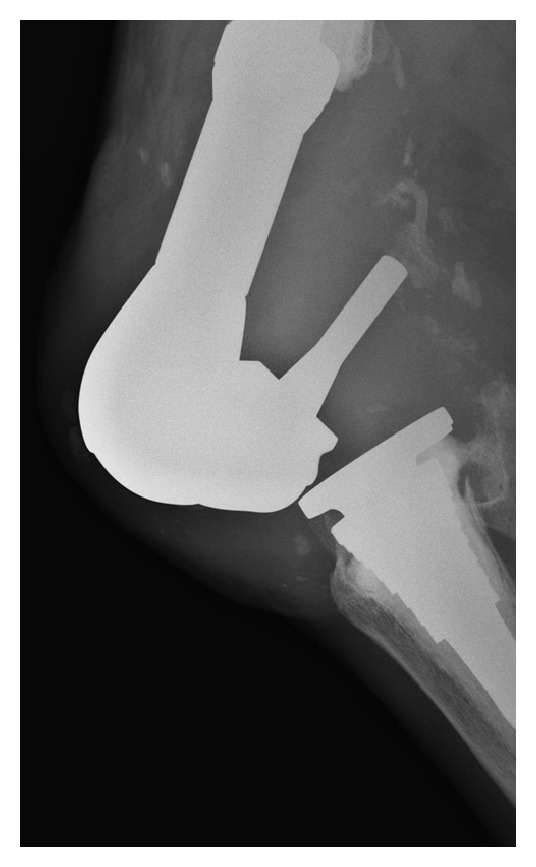
Preoperative lateral radiograph illustrating disengagement and posterior dislocation of the hinge post in close proximity to the location of the popliteal artery.

**Figure 2:**
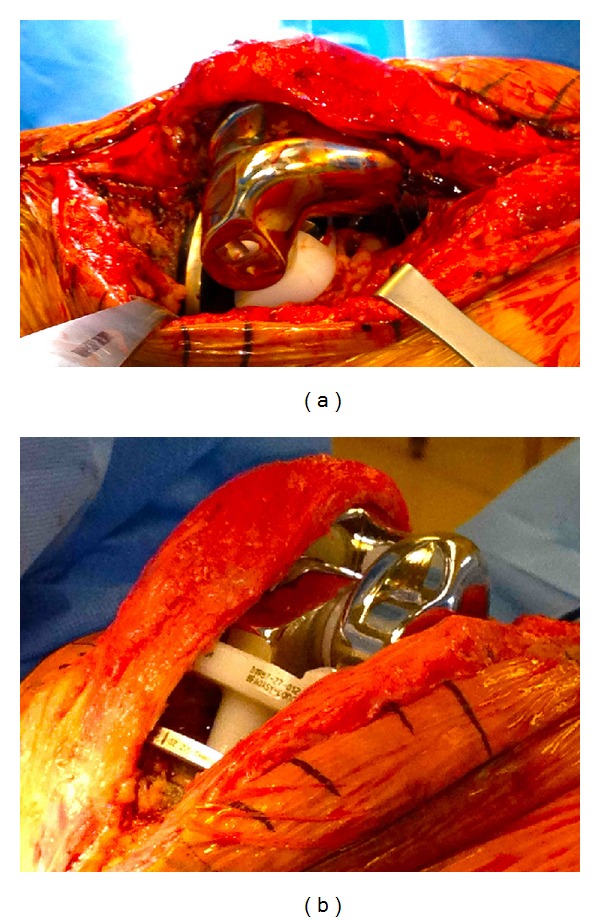
(a) Intraoperative findings illustrating dislocation of rotating hinge stem. (b) Intraoperative opening of the flexion gap causing dislocation.

**Figure 3:**
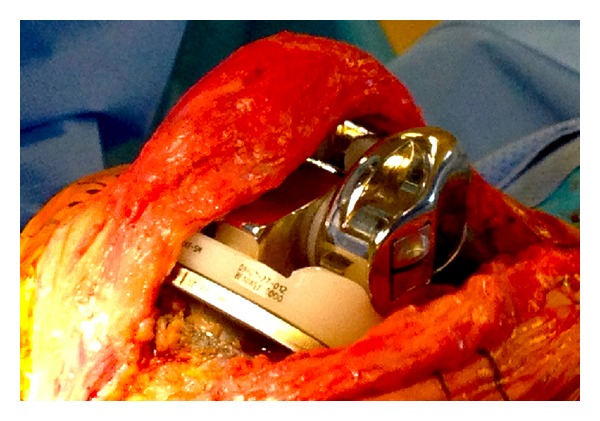
Intraoperative illustrating new tibial hinge insert, hinge pin, and locking pin.

P. K. is an 83-year-old male with a history of multiple health comorbidities and multiple revision right total knee arthroplasty (TKA) with a rotating hinge implant, who presented to our institution's emergency room with progressively worsening right knee pain causing instability and inability to ambulate after sustaining a fall while walking outdoors one month prior. The patient underwent total knee revision surgery 1 year prior at an outside institution where a DePuy S-ROM rotating hinge implant was used. Physical examination was limited due to gross deformity of the right knee. Neurovascular examination was intact with good distal pulses and an ankle brachial index >0.9. Radiographs revealed a posterior dislocation of the hinge post of the right total knee arthroplasty; the post was in close proximity to the location of the popliteal neurovascular structures (Figure 1). A lower extremity CT angiography showed intact vascular structures. Infection workup showed an increase in ESR and CRP however, fluid cultures and cell counts with differential from joint aspirate were unremarkable. Intraoperative findings confirmed a disengaged hinge that dislocated on flexion (Figures 2(a) and 2(b)) and a laterally dislocated patella. The mobile hinge components were removed and replaced with an extra-extra small-size bumper and a 12 mm tibial insert (Figure 3). Extensive lateral release was performed to centralize the laterally dislocated patella and restore proper patella tracking. Intraoperative arc of motion after revision of the hinge components was 0° to 90° against gravity, and no instability could be elicited. Postoperatively, the patient was placed in a hinged brace locked in full extension. At his one-month followup, he was ambulating painlessly with the assistance of a cane and demonstrated flexion up to 100°. Postoperative imaging showed a well-seated implant with no signs of loosening, fracture, dislocation, or wear. The patient was doing well on his 3-month and 1-year followup, ambulating with no assistive devices with good range of motion and no further instability and complications. The patient gave informed consent for the case to be published. 

## 3. Discussion

The use of rotating hinge knee prostheses has been suggested for use when constrained knee implants fail to provide proper stability. This is commonly seen in knees with deficient ligamentous structures secondary to tumor resection, trauma, multiple knee revisions, extensor mechanism dysfunction, distal femoral nonunion, or massive distal femoral bone loss [5, 6, 10, 15, 16]. Rotating hinge implants have been shown to improve pain [17–19] and range of motion [19, 20]; however, due to variable long-term outcomes, there is no universal agreement on its use in complex primary, revision, and salvage knee reconstruction [4, 5, 19, 21–25]. Despite evolution in their design, complication rates ranging from 0% to 44% at 3.8 to 15 years have been reported secondary to infection, aseptic loosening, patellar instability, and prosthetic dislocation [5, 26]. 

Mechanical dislocation of the rotating hinge knee prosthesis has been reported in the past due to breakage of the tibial hinge insert, yolk fracture, failure of the antidislocation feature, and breakage of the polyethylene bearing bush of the femoral channel component on the metallic tibial stud [7–9, 11, 12, 27]. However, pure dislocation of the hinge mechanism has only been published sparingly [10, 11]. In 2005, Ward and colleagues reported four cases of hinge dislocation in rotating hinge implants of varying designs [11]. Recently, Biswas and colleagues reported a case of atraumatic anterior disengagement of a hinge-post extension in a contemporary rotating hinge knee prosthesis [10]. 

Proposed mechanisms in the literature of pure atraumatic hinge dislocation include a combination of distraction-mediated disengagement, the screw home mechanism, and the rotating stem design [11, 28, 29]. The risk of tibiofemoral distraction-mediated disengagement is increased in patients with knee instability due to insufficient ligamentous support and balance and weakness of the joint capsule in cases of multiple revised total knee arthroplasty or rheumatoid knees [11]. Rapuri and colleagues suggested the screw home mechanism as an alternative cause of dislocation of the hinge post. They postulated that as lateral roll forward of the right knee occurs during extension, high rotational constraint, caused by the interaction of the femoral housing and the tibial post, produces a counterclockwise torque on the locking screw, which, over the years, may loosen and lead to subsequent disengagement of the hinge-post extension [28]. This counterclockwise torque was suggested to occur only in the right knee; however, reports of reverse screw home kinematics after TKA may allow loosening of the locking screw in the left knee to also occur [28]. Ward and colleagues conducted a biomechanical study evaluating design and function of various rotating hinge implants and suggested that the risk of developing instability and dislocation increases with a shorter rotating stem and greater taper [29]. They found that the DePuy Johnson & Johnson S-ROM rotating hinge required the least amount of distraction (26 mm) and had the greatest allowable angular tilt for dislocation to occur. By contrast, nontapered stems with cylindrical channels (Wright Medical Technology, Biomet, and Link America) or the long, minimally tapered Howmedica stem required the most distraction and had the least angular tilt for dislocation to occur [29]. Their subsequent report of four hinge dislocations led Ward and colleagues to suggest the use of a long, minimally tapered rotating stem to minimize chances of hinge stem dislocation. 

Opening of the flexion gap is suggested to be the mechanism of hinge dislocation in our patient. This was observed intraoperatively as passive flexion produced gross dislocation of the rotating hinge (Figures 2(a) and 2(b)). Failure of flexion gap stability and subsequent hinge dislocation occurred most likely from the patient's history of four right knee revisions, which increased the intrinsic laxity of knee soft tissues, and his lateral patellar dislocation, which compromised the extensor mechanism that normally functions to prevent the flexion gap from opening [30]. Patellofemoral instability is the most commonly reported complication after TKA and is a frequent source of postoperative morbidity such as dislocation of the rotating hinge stem [31]. The use of a short tapered rotating stem (DePuy S-ROM hinge) also helped facilitate the dislocation, requiring only 26 mm of distraction for disengagement to occur [29]. It is unlikely that the screw home mechanism played a role in the dislocation as noted by the tight-locking pins and screws observed intraoperatively. 

This experience exemplifies the importance of fundamental arthroplasty techniques such as proper soft tissue and gap balancing, adequate patellar tracking, restoration of the joint line, and repair of bone defects in achieving knee stability and minimizing dislocations in revision knee arthroplasty. The arthroplasty surgeon should be aware of the risk of dislocation of the rotating stem when stability cannot be achieved with soft tissue balancing. In these cases, we recommend the use of a long cylindrical, minimally tapered rotating stem as suggested by Ward and colleagues to decrease the risk of dislocation of the hinge.
